# Emergency surgery admissions and the COVID-19 pandemic: did the first wave really change our practice? Results of an ACOI/WSES international retrospective cohort audit on 6263 patients

**DOI:** 10.1186/s13017-022-00407-1

**Published:** 2022-01-28

**Authors:** Giovanni D. Tebala, Marika S. Milani, Mark Bignell, Giles Bond-Smith, Christopher Lewis, Roberto Cirocchi, Salomone Di Saverio, Fausto Catena, Marco Scatizzi, Pierluigi Marini, the CovidICE-International Collaborative, Rea Lo Dico, Antonio Stracqualursi, Giuseppe Russo, Sara D’Errico, Pasquale Cianci, Enrico Restini, Grazia Scialandrone, Gianluca Guercioni, Gennaro Martinez, Angela Pezzolla, Donato F. Altomare, Arcangelo Picciariello, Giuseppe Trigiante, Rigers Dibra, Vincenzo Papagni, Carolina Righetti, Roberto Polastri, Jacopo Andreuccetti, Giusto Pignata, Rossella D’Alessio, Elisa Arici, Ilaria Canfora, Nicola Cillara, Antonello Deserra, Raffaele Sechi, Francesco Bianco, Simona Gili, Antonio Cappiello, Paola Incollingo, Alan Biloslavo, Gabriele Bellio, Paola Germani, Nicolò De Manzini, Marco Buiatti, Fabio P. Paladino, Diego Sasia, Felice Borghi, Valentina Testa, Giorgio Giraudo, Fabrizio Allisiardi, Maria C. Giuffrida, Martino Gerosa, Alessandro Fogliati, Dario Maggioni, Nicolò Fabbri, Carlo V. Feo, Erica Bianchini, Ilaria Panzini, Vincenzo Lizzi, Fausto G. Tricarico, Giovanni Di Gioia, Rocco Melino, Nicola Tartaglia, Antonio Ambrosi, Giovanna Pavone, Mario Pacilli, Fernanda Vovola, Fiorenza Belli, Andrea Barberis, Antonio Azzinnaro, Andrea Coratti, Roberto Benigni, Stefano Berti, Michele Saracco, Andrea Gennai, Laura Dova, Roberto Farfaglia, Giacomo Pata, Valeria Arizzi, Giampiero Pandolfo, Alice Frontali, Piergiorgio Danelli, Luca Ferrario, Claudio Guerci, Nicolò M. Mariani, Andrea Pisani Ceretti, Vincenzo Nicastro, Enrico Opocher, Davide Gozzo, Gianmaria Casoni Pattacini, Maurizio Castriconi, Alfonso Amendola, Maria Gaudiello, Giuseppe Palomba, Fausto Catena, Gabriele L. Petracca, Gennaro Perrone, Mario Giuffrida, Gianluigi Moretto, Harmony Impellizzeri, Andrea Casaril, Mauro Filosa, Antonio Caizzone, Sonia Agrusti, Gaetano M. Cattaneo, Palomba Capelli, Andrea Muratore, Marcello Calabrò, Nicoletta S. Pipitore Federico, Bruno Cuzzola, Riccardo Danna, Alessandra Murgese, Federico Coccolini, Erica Pieroni, Massimo Chiarugi, Dario Tartaglia, Sandro Giannessi, Riccardo Somigli, Martina Trafeli, Massimo Fedi, Rosita De Vincenti, Anna Guariniello, Michele Grande, Giulia Bagaglini, Brunella Pirozzi, Andrea M. Guida, Sara Ingallinella, Cristine P. Don, Leandro Siragusa, Orazio Capone, Daniele Cerbo, Emanuele Santoro, Vito Pende, Alessia Fassari, Andrea Mingoli, Gioia Brachini, Bruno Cirillo, Martina Zambon, Pierfranco Cicerchia, Simona Meneghini, Paolo Sapienza, Antonella Puzzovio, Filippo La Torre, Pietro Fransvea, Marta Di Grezia, Gabriele Sganga, Mariano F. Armellino, Giovanna Ioia, Bernardino Rampone, Marcello Della Corte, Francesco Fleres, Guglielmo Clarizia, Pierpaolo Bordoni, Alessandro Spolini, Marco Franzini, Alessandro Grechi, Monica Suppo, Dario Bono, Donatella Scaglione, Christian Cotsoglou, Sissi Paleini, Andrea P. Chierici, Matteo Uccelli, Stefano Olmi, Giovanni Cesana, Nadia Tenreiro, Andre Marcal, Daniela Martins, Clara Leal, Bruno Vieira, Bakarne Ugarte-Sierra, Irune Vincene-Rodriguez, Marta Duran-Ballesteros, Amaia Sanz-Larrainzar, Francisco J. Ibanez-Aguirre, Carlos Yanez-Benites, Issa Talal, Juan L. Blas, Roberta Garau, Saskia Clark-Stuart, Angus Wallace, Andrew Di Carlo, Ellen Wisnia, Konain Ehsan, Kellen Beck-Sanders, Emma Godson, Paul Campbell, Giovanni D. Tebala, Mark Bignell, Giles Bond-Smith, Christopher Lewis, Raheel Ahmad, Roshneen Ali, Sambasivan S. Aswani, Afzal Barza, Catalina Carrillo, Aruna Dawani, Abishek Dey, Amr Elserafy, Diana Gaspar, Lucia Lazzareschi, Mitul Patel, Amanda Shabana, Mohamed Shams, Ola Shams, Zoe Slack

**Affiliations:** 1 Surgical Emergency Unit, Oxford University Hospitals NHS Foundation Trust, Oxford, UK; 2 Department of General Surgery, Causa Pia Luvini Hospital, Cittiglio, Italy; 3 Department of General Surgery, University of Perugia, Perugia, Italy; 4 Department of General Surgery, Madonna del Soccorso Hospital, San Benedetto del Tronto, Italy; 5 Department of General and Emergency Surgery, “M. Bufalini” Hospital, Cesena, Italy; 6 Department of General Surgery, S. Maria Annunziata Hospital, Firenze, Italy; 7 Department of General and Emergency Surgery, S. Camillo-Forlanini Hospital, Rome, Italy; 8 Consultant Emergency and Colorectal Surgeon, Oxford University Hospitals NHS Foundation Trust, John Radcliffe Hospital, Headley Way, Headington, Oxford, OX3 9DU UK

**Keywords:** Emergency surgery, Covid-19, Admission

## Abstract

**Introduction:**

The COVID-19 pandemic is having a deep impact on emergency surgical services, with a significant reduction of patients admitted into emergency surgical units world widely. Reliable figures of this reduction have not been produced yet. Our international audit aimed at giving a precise snapshot of the absolute and relative changes of emergency surgical admissions at the outbreak of the pandemic.

**Materials and methods:**

Datasets of patients admitted as general surgical emergencies into 45 internationally distributed emergency surgical units during the months of March and April 2020 (Covid-19 pandemic outbreak) were collected and compared with those of patients admitted into the same units during the months of March and April 2019 (pre-Covid-19). Primary endpoint was to evaluate the relative variation of the presentation symptoms and discharge diagnoses between the two study periods. Secondary endpoint was to identify the possible change of therapeutic strategy during the same two periods.

**Results:**

Forty-five centres participated sent their anonymised data to the study hub, for a total of 6263 patients. Of these, 3810 were admitted in the pre-Covid period and 2453 in the Covid period, for a 35.6% absolute reduction. The most common presentation was abdominal pain, whose incidence did not change between the two periods, but in the Covid period patients presented less frequently with anal pain, hernias, anaemia and weight loss. ASA 1 and low frailty patients were admitted less frequently, while ASA>1 and frail patients showed a relative increase. The type of surgical access did not change significantly, but lap-to-open conversion rate halved between the two study periods. Discharge diagnoses of appendicitis and diverticulitis reduced significantly, while bowel ischaemia and perianal ailments had a significant relative increase.

**Conclusions:**

Our audit demonstrates a significant overall reduction of emergency surgery admissions at the outbreak of the Covid-19 pandemic with a minimal change of the proportions of single presentations, diagnoses and treatments. These findings may open the door to new ways of managing surgical emergencies without engulfing the already busy hospitals.

## Introduction

The Covid-19 pandemic has radically changed our life and work. In particular, it is having a significant and long-lasting impact on healthcare [1]. Each and every health system underwent radical changes to adapt to the growing number of emergency admissions for respiratory syndromes, most of which needed intensive care, and every Country produced their own guidelines and policies to deal with this unexpected disaster [2]. Most elective services have been stopped, with some differences among Countries, but emergency admissions had to be treated anyway, although with reduced resources. A common government advice was to avoid attending the Emergency Department if not strictly necessary, to leave room for more severe cases of Covid-19-associated respiratory failure and to avoid Covid-19 cross-infection. This caused a perceived reduction of all the non-Covid emergency admissions, in particular those of surgical remit. However, the general perception among surgeons was that the many causes for emergency presentation did not reduce proportionally. Aim of our international audit was to have a snapshot of the absolute and relative change of surgical emergency admissions during the first wave of the Covid-19 pandemic. Limiting our audit to the outbreak of the pandemic—instead of considering the whole course—allowed us to analyse the impact of the worldwide infection at its maximum, when most Countries were not yet prepared to respond.

## Materials and methods

We analysed data of patients admitted into Emergency Surgery Units in the months of March and April 2020 and compared them with same datasets of patients admitted in the months of March and April 2019. March–April 2020 were the first two months of the global pandemic in Europe. The control period March–April 2019 was chosen as a significant match to avoid seasonal biases.

An invitation letter with a short version of the protocol of this retrospective audit was disseminated by email to more than 6000 surgeons in Europe using the ACOI and WSES mailing lists. The units that confirmed their interest in participating to this study were emailed the full protocol and an empty database to collect their anonymised demographic and clinical data. Only adult patients admitted to the Surgical Emergency Units have been included. Patients aged < 18 and those with < 80% of complete data have been excluded. Single patients’ data have been accessed only by those who have anyway daily access to that information for their clinical work. Data from each unit have been collected under the responsibility of the Principal Local Investigator (PLI), anonymised and sent by secure encrypted email to the Study Coordinator (SC). Thereafter, data have been centrally coded, collected into an encrypted electronic database (Microsoft Excel for Mac v.16.54) and analysed with a statistical package (StatPlus Mac Pro 8.0.1.0). No patient could be identified in the main central database.

Frequency variables have been compared by the Pearson Chi-square test. Continuous variables have been tested for distribution and skewness and then compared by one-way ANalysis Of VAriance (ANOVA). Values of *p* < 0.05 have been considered significant.

Primary endpoint of the study was to evaluate the relative variation of the presentation characteristics and discharge diagnoses between the two study periods.

Secondary endpoint was to identify the possible change of therapeutic strategy during the same two periods.

Independent variable was the year of presentation (March-April 2019 vs March-April 2020).

Analysed dependent variables were: age, gender, presentation, smoking rate, ASA, frailty score, treatment, surgical access, lap-to-open conversion, surgical subspecialty, length of stay, reoperation rate, readmission rate (Tables 1 and 2).

**Table 1:** Characteristics of the patients and variations between the two periods of the study

Factor	2019*	2020*	Absolute variation§	*P§*
**Total**	3810	2453	− 35.6%	
Age	59.1 ± 21.1	59.9 ± 20.3	+ 1.4%	0.11676
Gender (F/M)	1875/1935	1211/1242	− 35.4%/− 35.8%	0.90435
**Presentation**				
Abdominal pain	2709 (71.1%)	1680 (68.5%)	− 38.0%	**0.00552**
Bowel obstruction	183 (4.8%)	148 (6.0%)	− 19.1%	
GI bleeding	154 (4.0%)	142 (5.8%)	− 7.8%	
Anorexia/Weight loss	7 (0.2%)	3 (0.1%)	− 57.1%	
Jaundice	113 (3.0%)	67 (2.7%)	− 40.7%	
Diarrhoea/Vomit	92 (2.4%)	63 (2.6%)	− 31.5%	
Dysphagia	9 (0.2%)	10 (0.4%)	+ 11.1	
Non-trauma haemoperitoneum	4 (0.1%)	2 (0.1%)	− 50.0%	
Anal pain	114 (3.0%)	49 (2.0%)	− 57.0%	
Fever	24 (0.6%)	19 (0.8%)	− 20.8%	
Anaemia	15 (0.4%)	4 (0.2%)	− 73.3%	
Hernia	169 (4.4%)	94 (3.8%)	− 44.4%	
Skin issue	60 (1.6%)	51 (2.1%)	− 15.0%	
Wound complications	10 (0.3%)	2 (0.1%)	− 80%	
Postop complications	43 (1.1%)	34 (1.4%)	− 20.9%	
Breast pain	5 (0.5%)	7 (0.3%)	+ 40.0%	
Chest pain	40 (1.0%)	26 (1.1%)	− 35%	
Ischaemic limb	12 (0.3%)	12 (0.5%)	0%	
Non traumatic shock	17 (0.4%)	17 (0.7%)	0%	
Abnormal radiology	6 (0.2%)	2 (0.1%)	− 66.7%	
Other	24 (0.6%)	21 (0.9%)	− 12.5%	
*Missing*	*0*	*0*		
**Smoking**				
No	2616 (72.7%)	1428 (61.5%)	− 45.4%	**< 0.0001**
Yes	833 (23.2%)	599 (25.8%)	− 28.1%	
Ex	147 (4.1%)	296 (12.7%)	+ 101.4%	
*Missing*	*214*	*130*		
**ASA**				
1	1007 (29.0%)	477 (20.6%)	− 52.6%	**< 0.0001**
2	1167 (33.6%)	834 (36.0%)	− 28.5%	
3	1004 (28.9%)	749 (32.4%)	− 25.4%	
4	277 (8.0%)	238 (10.3%)	− 14.1%	
5	16 (0.5%)	16 (0.7%)	0%	
*Missing*	*339*	*139*		
**Frailty score**				
1-2	2060 (54.1%)	1165 (47.5%)	− 43.4%	**< 0.0001**
>2	1750 (45.9%)	1287 (52.5%)	− 26.5%	
*Missing*	*0*	*1*		

^*^: values are reported as: total number (relative percentage in the column)

^§^: values represent the absolute variation of cases between March-April 2019 and March-April 2020

§: in bold* p* < 0.05

**Table 2:** Treatment of the patients and its outcome and variations between the two periods of the study

Factor	2019*	2020*	Absolute variation§	*P*
**Treatment**				
Medical	1175 (30.8%)	808 (33.0%)	− 31.2%	0.12282
Surgical	2391 (62.8%)	1505 (61.4%)	− 37.1%	
Interventional	244 (6.4%)	137 (5.6%)	− 43.9%	
*Missing*	*0*	*3*		
**Surgical access**				
Laparoscopic	952 (42.7%)	559 (40.4%)	− 41.3%	0.11324
Open	1257 (56.4%)	818 (59.1%)	− 34.9%	
Lap converted to open	19 (0.8%)	6 (0.4%)	− 68.4%	
*Missing*	*171*	*107*		
Lap-to-open conversion	19/971 (2.0%)	6/565 (1.1%)	− 45.0%	0.18140
**Surgical subspecialty**				
Colorectal	1307 (54.9%)	809 (53.9%)	− 38.1%	**0.00603**
Upper GI	85 (3.6%)	70 (4.7%)	− 17.6%	
HPB	416 (17.5%)	256 (17.1%)	− 38.5%	
Abdominal wall	281 (11.8%)	150 (10.0%)	− 46.6%	
I&D of abscesses	138 (5.8%)	98 (6.5%)	− 29.0%	
Chest	35 (1.5%)	13 (0.9%)	− 62.9%	
Vascular	21 (0.9%)	11 (0.7%)	− 47.6%	
Urology/Gynae	14 (0.6%)	7 (0.5%)	− 50.0%	
Other	82 (3.4%)	87 (5.8%)	+ 6.1%	
*Missing*	*12*	*4*		
Length of stay	7.8±10.5	7.7±10.0	− 1.5%	0.65716
**Reoperation**				
No	3457 (96.6%)	2334 (96.6%)	− 32.5%	0.93414
Yes	120 (3.4%)	82 (3.4%)	− 31.7%	
*Missing*	*233*	*37*		
**Readmission**				
No	3398 (91.0%)	2247 (92.4%)	− 33.9%	**0.05906**
Yes	335 (9.0%)	185 (7.6%)	− 44.8%	
*Missing*	*77*	*21*		

^*^: values are reported as: total number (relative percentage in the column)

^§^: values represent the absolute variation of cases between March-April 2019 and March-April 2020

§: in bold* p* < 0.05

Missing data have been excluded listwise. This may account for minimal numeric discrepancies in the calculation of frequencies. The factors who had more than 10% of missing data have been excluded from the analysis.

Values in the text, tables and figures are approximated to the tenths. *P*-values are not approximated.

Ethical committee approval was not considered to be necessary due to the retrospective nature of the study and the fact that sensible data were all fully anonymised by the local research team before being transmitted and analysed by the central team. However, the study was approved by the Comitato Etico ATS Sardegna on 22.12.2020 and was approved and endorsed by the World Society of Emergency Surgery (WSES) and the Association of Italian Hospital Surgeons (ACOI—Associazione dei Chirurghi Ospedalieri Italiani).

This paper has been drafted according to the STrengthening the Reporting of OBservational studies in Epidemiology (STROBE) checklist [3].

## Results

Forty-five centres participated in the study (39 from Italy, 2 from UK, 2 from Spain, 1 from Portugal, 1 from France) for a total of 6263 patients.

**Fig. 1:**
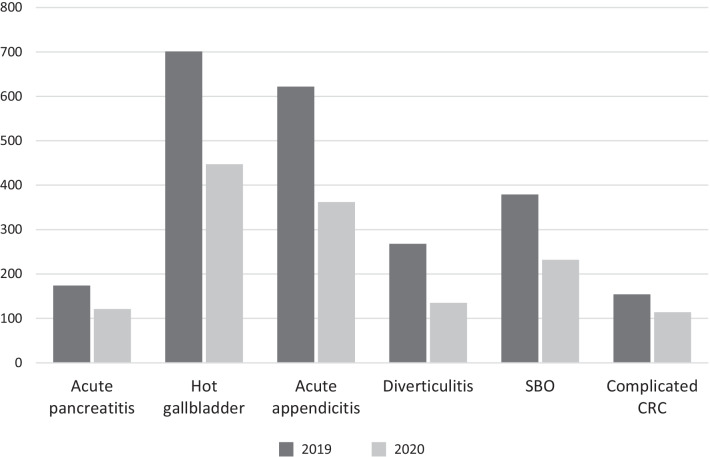
Most common diagnoses (*p* = 0.23745). “Hot gallbladder” includes acute cholecystitis and refractory biliary colic. “Diverticulitis” includes complicated and uncomplicated acute diverticulitis. “SBO” is small bowel obstruction. “CRC” is colorectal cancer

Results are listed in Tables 1, 2, 3, 4, 5 and in Figure 1. Only the most significant are hereby reported.

**Table 3:** Surgical access for the most common operations

	Access	2019	2020	*P*
Appendicectomy	Laparoscopic	434 (81.6%)	249 (83.0%)	0.36888
	Open	91 (17.1%)	50 (16.7%)	
	Lap to open	7 (1.3%)	1 (0.3%)	
Cholecystectomy	Laparoscopic	321 (81.9%)	188 (76.4%)	0.24021
	Open	68 (17.3%)	56 (22.8%)	
	Lap to open	3 (0.8%)	2 (0.8%)	
Hernia repair*	Laparoscopic	22 (7.8%)	12 (8.1%)	0.91902
	Open	259 (92.2%)	136 (91.9%)	
	Lap to open	0	0	
Large bowel resection	Laparoscopic	40 (14.6%)	24 (11.6%)	0.49189
	Open	231 (84.6%)	180 (87.4%)	
	Lap to open	2 (0.7%)	2 (1.0%)	
Small bowel resection	Laparoscopic	3 (1.8%)	4 (3.0%)	0.57322
	Open	165 (96.5%)	122 (96.1%)	
	Lap to open	3 (1.8%)	1 (0.8%)	

^*^ “Hernia repair” includes inguinal, femoral, umbilical, ventral and incisional hernias

**Table 4:** Discharge diagnosis in the two periods of the study

Diagnosis	2019*	2020*	Absolute variation§	*P§*
Acute appendicitis	609 (16.0%)	356 (14.5%)	− 41.5%	**< 0.0001**
Acute cholecystitis	445 (11.7%)	310 (12.6%)	− 30.3%	
Small bowel obstruction	379 (9.9%)	232 (9.5%)	− 38.8%	
Complicated diverticulitis	268 (7.0%)	135 (5.5%)	− 49.6%	
Complicated inguinal hernia	199 (5.2%)	110 (4.5%)	− 44.7%	
Acute pancreatitis	174 (4.6%)	121 (4.5%)	− 30.5%	
Complicated colon cancer	155 (4.1%)	114 (4.5%)	− 26.5%	
Complicated gastritis/peptic ulcer	134 (3.5%)	88 (3.6%)	− 34.2%	
Complicated ventral hernia	132 (3.5%)	84 (3.4%)	− 36.4%	
CBD stones	129 (3.4%)	76 (3.1%)	− 41.1%	
Gallstones	124 (3.3%)	61 (2.5%)	− 50.8%	
Non-specific abdominal pain	112 (2.9%)	71 (2.9%)	− 36.6%	
Perianal abscess/fistula/fissure	101 (2.7%)	69 (2.8%)	− 31.7%	
Superficial collection	66 (1.7%)	52 (2.1%)	− 21.2%	
Advanced cancer (any)	62 (1.6%)	28 (1.1%)	− 54.8%	
Bowel ischaemia	56 (1.5%)	51 (2.1%)	− 8.9%	
Constipation	45 (1.2%)	9 (0.4%)	− 80%	
Small bowel cancer	38 (1.0%)	30 (1.2%)	− 21.1%	
UTI/orchitis/epididymitis/torsion	30 (0.8%)	20 (0.8%)	− 33.3%	
Postop bleeding	30 (0.8%)	1 (0.04%)	− 96.7%	
Upper GI cancer	30 (0.8%)	17 (0.7%)	− 43.3%	
Haemorrhoids/Prolapse	28 (0.7%)	26 (1.1%)	− 7.1%	
Ischaemic limb	28 (0.7%)	21 (0.5%)	− 57.1%	
Spontaneous PNX	28 (0.7%)	12 (0.5%)	− 57.1%	
Pancreatic cancer	25 (0.7%)	8 (1.2%)	− 68.0%	
Anastomotic leak	24 (0.6%)	19 (0.8%)	− 0.8%	
Sigmoid/Caecal volvulus	22 (0.6%)	17 (0.7%)	− 22.7%	
GORD / Hiatus hernia	22 (0.6%)	11 (0.4%)	− 50.0%	
Goitre	22 (0.6%)	20 (0.8%)	− 9.1%	
Anaemia	22 (0.6%)	21 (0.9%)	− 4.5%	
IBD	20 (0.5%)	22 (0.9%)	+ 10.0%	
Deep SSI	18 (0.5%)	6 (0.2%)	− 66.7%	
Abdominal abscess	18 (0.5%)	19 (0.8%)	+ 5.6%	
Stoma complication	16 (0.4%)	43 (1.8%)	+ 168.8%	
Spontaneous haemoperitoneum	16 (0.4%)	6 (0.2%)	− 62.5%	
Iatrogenic GI perforation	13 (0.3%)	9 (0.4%)	− 30.8%	
Hepatobiliary cancer	11 (0.3%)	7 (0.3%)	− 36.4%	
Benign large bowel obstruction	10 (0.3%)	10 (0.4%)	0%	
Biliary fistula	8 (0.2%)	2 (0.1%)	− 75.0%	
Superficial SSI	8 (0.2%)	6 (0.2%)	− 25.0%	
Kidney stone	8 (0.2%)	3 (0.1%)	− 62.5%	
PID / Endometriosis	8 (0.2%)	3 (0.1%)	− 62.5%	
Medical sepsis	7 (0.2%)	3 (0.1%)	− 57.1%	
Unspecified peritonitis	5 (0.1%)	2 (0.1%)	− 60.0%	
Breast infection	5 (0.1%)	6 (0.2%)	+ 20.0%	
Aortic aneurysm	4 (0.1%)	2 (0.1%)	− 50.0%	
Enteric fistula	4 (0.1%)	6 (0.2%)	+ 50.0%	
Liver abscess	4 (0.1%)	4 (0.2%)	0%	
GIST	4 (0.1%)	4 (0.2%)	0%	
Oesophageal varices	3 (0.1%)	4 (0.2%)	+ 33.3%	
Small bowel perforation	3 (0.1%)	0	− 100.0%	
Small bowel/large bowel bleeding	3 (0.1%)	3 (0.1%)	0%	
Upper GI stricture	2 (0.1%)	23 (0.9%)	+ 1050.0%	
Meckel	2 (0.1%)	1 (0.04%)	− 50.0%	
Spleen/liver ischaemia, bleeding, cyst	2 (0.1%)	6 (0.2%)	− 200.0%	
Gallstone ileus	2 (0.1%)	0	− 100.0%	
Infective colitis/enteritis	0	1 (0.04%)	+ 100.0%	
Bladder perforation	0	1 (0.04%)		
Other	67 (1.8%)	70 (2.8%)	+ 4.5%	
*Missing*	*0*	*0*		

^*^: values are reported as: total number (relative percentage in the column)

^§^: values represent the absolute variation of cases between March-April 2019 and March-April 2020

§: in bold* p* < 0.05

**Table 5:** Treatment per diagnosis per year (most common diagnoses)

	Treatment	2019	2020	*P§*
Acute pancreatitis	Medical	132 (75.9%)	96 (79.3%)	0.48247
	Surgical	27 (15.5%)	19 (15.7%)	
	Interventional	15 (8.6%)	6 (5.0%)	
Hot gallbladder	Medical	202 (28.8%)	136 (30.6%)	0.81865
	Surgical	401 (57.2%)	248 (55.7%)	
	Interventional	98 (14.0%)	61 (13.7%)	
Acute appendicitis	Medical	53 (8.5%)	32 (8.8%)	0.97813
	Surgical	567 (91.2%)	329 (90.9%)	
	Interventional	2 (0.3%)	1 (0.3%)	
Diverticulitis	Medical	159 (59.3%)	73 (54.1%)	0.49189
	Surgical	99 (36.9%)	58 (43.0%)	
	Interventional	10 (3.7%)	4 (3.0%)	
Small bowel obstruction	Medical	131 (34.6%)	103 (44.4%)	**0.02324**
	Surgical	241 (63.6%)	128 (55.2%)	
	Interventional	7 (1.8%)	2 (0.5%)	
Complicated colorectal cancer	Medical	12 (7.8%)	8 (7.0%)	**0.04585**
	Surgical	131 (85.1%)	105 (92.1%)	
	Interventional	11 (7.1%)	1 (0.9%)	

§: in bold* p* < 0.05

Table 1 reports the characteristics of the patients enrolled in the study. In March-April 2019 the participating units admitted 3810 patients, whereas during the Covid-19 first wave they admitted 2453 patients, for a 35.6% absolute reduction. Therefore, in evaluating the reduction of single items, a reduction of or around 35.6% is considered to be due to the overall reduction of admissions, whereas a variation of more than -35.6% may signify a relative reduction of that single factor and a reduction of less than -35.6% means that that single factor had a relative increase. In case of positive variation, it means that that item has had a significant relative increase.

Age of admitted patients and gender distribution did not change between the two periods (59.1 ± 21.1 vs. 59.9 ± 20.3, *p* = NS, and 1875/1935 vs. 1211/1242, *p* = NS, respectively). The most frequent reason for admission was “abdominal pain” in both periods, with a reduction of 38%. This is still consistent with the general reduction of admissions. However, some causes of presentation reduced much more than expected. This is the case with “anal pain” (− 57%), “anaemia” (− 73.3%), “hernias” (− 44.4%), “anorexia/weight loss” (− 57.1%) and “wound complications” (-80%). Other presentations reduced much less or, unexpectedly, increased, such as “bowel obstruction” (− 19.1%), “GI bleeding” (− 7.8%) and “dysphagia” (+ 11.1%).

The number of ASA 1 patients decreased of 52.6%, while patients in poorer general conditions were admitted more often (variation: ASA 2, − 28.5%, ASA 3, − 25.4%, ASA 4, − 14.1%, ASA 5, 0%). This trend has been confirmed by evaluating the Frailty Score. Patients with no or minimal frailty (1–2) were admitted less frequently (− 43.4% reduction between 2019 and 2020), whereas more frail patients were admitted more frequently (− 26.5% reduction, which means a relative increase with respect to the − 36.5% overall reduction of admissions).

Table 2 shows the treatment of the patients and their outcomes. The type of treatment (medical vs. surgical vs. interventional) and the surgical access (laparoscopic vs. open vs. laparoscopic converted to open) did not change much during the two study periods. However, the rate of laparoscopy-to-open conversion halved between the two periods (not statistically significant, though).

To assess if the unbalanced distribution of participating units would add a significant bias, a comparison between Italian and non-Italian centres has been performed. It showed that Italian surgeons were more prone to treat their patients with surgery in both periods with respect to their non-Italian counterparts (in 2019, 68.5% vs. 48.7%, in 2020, 70.7% vs. 42.6%, *p* = 0.000). Furthermore, while non-Italian centres showed a small relative reduction of the surgical approach (from 48.7% of all treatments to 42.6%, variation = − 12.6%) (and a contemporaneous increase of non-operative management), the attitude of Italian units showed a minimal relative increase in the surgical approach (from 68.5% of all treatments to 70.7%, variation = + 3.2%), but this difference was not statistically significant.

In the general series, some subspecialties had a more significant reduction (*p* = 0.006). For instance, chest and abdominal wall surgery reduced more (respectively, − 62.9% and − 46.6%), while upper GI had a relative increase (− 17.6%).

Total length of stay was 7.8 ± 10.5 days in the first period and 7.7 ± 10.0 days in the second period (*p* = NS). The percent reduction of the reoperations was consistent with the overall reduction of cases (*p* = NS), but reduction of readmissions was significantly more than expected (− 44.8%, *p* = 0.059).

Table 3 examines the surgical access in the most common operations. Appendicectomies and cholecystectomies were performed by laparoscopy in the vast majority of cases (81.6% in 2019 and 83.0% in 2020 and 81.9% in 2019 and 76.4% in 2020, respectively). Hernia repairs, large bowel resections and small bowel resections have been more frequently performed by open surgery (92.2% vs. 91.9%, 84.6% vs. 87.4% and 96.5% vs. 96.1%, respectively). There was no significant difference of approach between the two periods of the study.

Tables 4 and 5 and Figure 1 deal with discharge diagnosis. The most frequent discharge diagnoses were acute appendicitis, acute cholecystitis and small bowel obstruction (SBO). The overall incidence of the different diagnoses changed significantly between the two study periods (*p* < 0.0001). While the incidence of admission for “acute appendicitis” reduced by 41.5% (much more than expected) and for “acute diverticulitis” reduced by 49.6%, other diagnoses, such as “bowel ischaemia” and “haemorrhoids/rectal prolapse” reduced less significantly (respectively, − 8.9% and − 7.1%), thus showing a relative growth, and others even had an absolute increase (IBD + 10%, stoma complications + 168.8%, enteric fistulas + 50%, oesophagogastric strictures + 1050%). An analysis of the most frequent diagnoses (acute pancreatitis, hot gallbladder, acute appendicitis, diverticulitis, small bowel obstruction and complicated colorectal cancer) whose results are depicted in Fig. 1, did not show any relative change.

The treatment of acute pancreatitis, hot gallbladder, acute appendicitis and diverticulitis did not change significantly, but SBO was treated surgically much less frequently (63.6% in 2019, 55.2% in 2020—*p* = 0.02324) and complicated colorectal cancer much more frequently (85.1% vs. 92.1%—*p* = 0.04585).

## Discussion

Covid-19 is a worldwide pandemic affecting all Countries and taking by surprise every national healthcare system at its onset. Fortunately, due to immunization policies and containment guidelines, possibly helped by the natural weakening of the virus, the second and third waves of infection have been slightly less severe than the first and thankfully now the infection is reducing, at least in the western Countries. In most cases, social isolation has been used to control the spread of the virus. In some Countries, like the UK, this has meant an almost complete cessation of elective surgical work with significantly reduced capacity within primary care due to a combination of strict triage of patients and sickness within the workforce. Other governments and scientific societies have produced different guidelines, leading to different outcomes, mostly regarding the reduction of elective surgical operations. Sometimes, medical guidelines have been too drastic and widely controversial [1, 2] also because with the aim of creating room for Covid-19 patients they reduced the capacity for elective and non-Covid emergency patients. On the contrary, we believe, in agreement with other Authors [4], that therapeutic indications should not change during this pandemic, because in our role of doctors in “developed” Countries we are morally bound to maintain high standards of care despite the pandemic [5]. It would be expected that this reduction in capacity would result in an increase in attendances across emergency services within secondary care, but this has failed to materialize with a reduction in attendances in all emergency departments including surgical emergency units.

Our audit demonstrated an overall reduction in surgical emergency admission of about 36%, while average age and gender distribution did not change significantly. Few Italian Authors have reported a much more significant reduction on the basis of their own local experience [6–8], while a study from Australia reported a minimal reduction in overall emergency cases of only 12%, and a subgroup analysis of the general surgery emergencies shows a variation of − 14% [9]. These disparities can be due to the different national guidelines, but the variations between the pre-Covid era and the first months of the Covid outbreak always present with a minus sign.

The reason for this general reduction of admissions, that reflects a general reduction of presentations to the Emergency Departments, is not utterly clear, but it may be due to the government policies of self-isolation with the aim to avoid overstretching the health system or probably just to the fear of being infected while attending the hospital [7]. However, it is not clear also if this reduction is associated with an actual decrease of the incidence of emergency pathologies—possibly explained by a healthier lifestyle during lockdown—or if it is because patients with fewer symptoms and no systemic illness decided to stay at home and possibly self-medicate. In this last case, we would expect that the admitted patients were in poorer general conditions. In fact, our data showed a significant reduction of ASA 1 patients (− 52%), while ASA 5 patients did not reduce at all. Also in percentage, ASA 1 patients passed from 29 to 21% of all admitted patients, while ASA 3 and 4 patients passed from 29% and 8% to 32% and 10% respectively. Same results were found by analyzing the frailty score, where score 1-2 patients went from 54 to 48% and score > 2 went from 46 to 53%. Expectedly, these findings confirm that patients in poorer general conditions were more likely to attend the hospital during lockdown with respect to healthier ones.

It could be surely argued that the suggestion to “stay at home as much as possible” to save precious resources for more severe Covid patients might have caused the presentation of patients in more advanced stages of the disease. This may possibly be the case with acute cholecystitis and acute appendicitis [10] and more generally with peritonitis [11]. Our study did not consider the degree of severity of each of the analysed conditions, but if we take the therapeutic approach as an indirect index of severity, we could state that in our experience there was no difference in severity of the presentations between the pre-Covid and the Covid eras (Table 5).

The indications for surgery did not change during the first lockdown, despite suggestions and guidelines. However, this may be a biased conclusion, because most of the patients included into the analysis are from Italy and in that Country the guidelines were not so restrictive as elsewhere, where they suggested conservative non-operative management as much as possible against surgery. In fact, our data suggest that Italian surgeons were more aggressive in that they tended to use surgery much more frequently than their non-Italian colleagues in both periods of the study, with no change of attitude during the pandemic, while non-Italian units became even more conservative. This difference is not statistically significant and does not affect the overall results of our audit but shows a different cultural approach to surgical emergencies in terms of different criteria of admissions—where Italian surgeons tend to admit patients who more likely would need surgery—or different choices of treatment strategy—where non-Italian units, possibly considering local factors, prefer conservative management as much as possible.

Interesting data came from the observation that patients with small bowel obstruction (SBO) were treated with surgery much less frequently during the pandemic, thus confirming the suggestion of evidence and guidelines [12]. While other diagnoses, such as appendicitis, are generally not a therapeutic dilemma, our audit showed that our treatment of patients with SBO may be too often too aggressive. In fact, while in the pre-Covid era patients with SBO were treated by surgery still in almost 64% of cases, during the Covid lockdown surgery was considered necessary only in 55% of patients (Table 5). We believe that this percentage can be further reduced as soon as the above reported results are incorporated in new policies and guidelines on the treatment of SBO possibly reinforcing the role of non-operative management. On the contrary, the treatment of acute diverticulitis and complicated colorectal cancers was much more frequently surgical during the pandemic than before it, probably reflecting a late presentation of those patients due to Government advice to stay at home as much as possible to avoid impacting on the already busy hospitals.

The kind of access (laparoscopic vs. open vs. laparoscopic converted to open) did not change significantly, but the comparative analysis showed that during the first Covid lockdown 40% were treated laparoscopically, against 43% of the pre-Covid period, showing a 5% relative reduction. Interestingly, there were less lap-to-open conversions during the Covid period, probably as an unwanted consequence of restricted indications to laparoscopy, as some hasty guidelines suggested. These results were confirmed also at the subgroup analysis by type of operation for the most common procedures (Table 3). Many Authors suggested avoiding laparoscopy on the ground that it could be considered “unsafe” due to the risk of inhalation of surgical plume. We agree that the inhalation of surgical smoke can potentially be an occupational health issue due to chemical risk, but in the specific situation of the Covid pandemic, there is still no clear evidence suggesting transmission of viral particles through the surgical smoke [13].

Probably a stricter selection of patient led to a reduced rate of readmission (overall -16%, from 9% to less than 8%), but this can also be due to the gradual introduction, during the first lockdown, of social restrictions in terms of more difficult access to hospital care, so that patients who previously would be admitted for a postoperative complication, were preferably treated at home during the Covid lockdown.

The distribution of the various discharge diagnosis changed significantly during the Covid period with respect to the pre-Covid era (Table 4). Some of the changes we have highlighted during our audit can be due to normal variations, but some other are clearly not random but somehow related to the Covid pandemic or, better, to our response to it. This is for instance the case with acute appendicitis and acute diverticulitis, that had an absolute reduction of 42% and 50% respectively. As previously discussed, it is not clear if this reduction reflects a true fall of the incidence of these disorders or if it is due to a strict selection of the patients to be admitted, while less severe cases could be treated at home. This should open a frank discussion aimed at reviewing our criteria of surgical admission. In fact, if we could demonstrate that more patients can be easily treated at home with the same results of in-patients, we would introduce a huge improvement of resource utilization in our already very stretched national health systems. Initiative like the “Hospital At Home” scheme should be widely applied also to surgical emergency patients [14].

Other diseases likely reduced due to more people staying at home, and therefore hopefully having better diets and lifestyles, as it may be the case with symptomatic gallstones and gastro-oesophageal reflux, both reduced by more than 50%.

On the contrary, other diagnoses became more frequent. We confirmed the common perception that the incidence of symptomatic haemorrhoids and rectal prolapse increased significantly during the first lockdown. This can be explained with the more sedentary life and the reduction of physical workout. On the contrary, the significant increase of stoma complications (+168.8%) demonstrated in our audit may be a direct consequence of the temporary closure of non-vital health services. A much more extensive investigation would be necessary to explain the relative increase of cases of bowel ischaemia as it is well known that Covid infection can cause microvascular thrombosis [15], but an increase of cases of unexplained bowel ischaemia during the Covid period has been demonstrated also in patients who resulted negative at naso-pharyngeal swab and broncho-alveolar lavage [16]. This in-depth analysis is beyond the scope of this work.

Significant strength of this paper is its wide sample size due to its multicentric spread, yielding clear and significant data on emergency surgical admissions during the first wave of Covid-19 pandemic as compared with the same period of the previous year. The characteristics of admitted patients have been reported and analysed thoroughly.

This paper has also some limitations. First, it is supposed to give only a snapshot of the first two months of pandemic, but the Covid-19 infection did not break out at the same time in different Countries, therefore the choice of March-April may be arbitrary. Second, the fact that most of participating centres were in Italy might have introduced a selection bias linked to the different national policies and guidelines, but, as discussed above, this is not statistically significant.

## Conclusions

In conclusion, this paper demonstrates a significant overall reduction of emergency surgical admissions during the first lockdown, with a minimal but significant change of the proportions of the single presentations and diagnoses. Further large studies may be necessary to find out if the reduction of surgical emergency admissions reflects a true reduction of incidence or it is just the evidence that more patients were treated non-operatively at home with the same results than during an hospital admission.

Assuming that the reduction of admissions is due to more patients being treated at home, this opens the door to new ways to manage surgical emergencies while reducing the pressure on the already stretched resources of our health systems. Clearly, new policies in this sense must be supported by a growing cooperation between patients (= customers), health professionals and policy-makers, all committed towards a more sustainable and evidence-based healthcare.

## Data Availability

The dataset generated and analysed during the current study are available from the corresponding Author upon reasonable request.

## Abbreviations

ACOI: Associazione dei Chirurghi Ospedalieri Italiani (Association of Italian Hospital Surgeons)
ANOVA: Analysis of variance
ASA: American Society of Anaesthesiologists
NS: Not significant
GI: Gastrointestinal
IBD: Inflammatory bowel disease
PI: Principal investigator
PLI: Principal local investigator
SBO: Small bowel obstruction
SC: Study coordinator
STROBE: Strengthening the reporting of observational studies in epidemiology
WSES: World Society of Emergency Surgery

## References

[1] Guidance for health system contingency planning during widespread transmission of SARS-CoV-2 with high impact on healthcare services. https://www.ecdc.europa.eu/sites/default/files/documents/COVID-19-guidance-health-systems-contingency-planning.pdf. Last accessed 22 Dec 2021.

[2] Tebala GD, Bond-Smith G (2020). Guidelines and recommendations during the Covid-19 pandemic: a word of caution. Am J Surg.

[3] von Elm E, Altman DG, Egger M, Pocock SJ, Gøtzsche PC, Vandenbroucke JP (2008). STROBE initiative. The strengthening the reporting of observational studies in epidemiology (STROBE) statement: guidelines for reporting observational studies. J Clin Epidemiol.

[4] Aranda-Narvaez JM, Tallon-Aguilar L, Pareja-Ciurò F, Martin-Martin G, Gonzales-Sanchez AJ, Rey-Simo I, Tamayo-Medel G, Yanez-Benitez C, Costa-Navarro D, Monton-Condon S, Navarro-Soto S, Turegano-Fuentes F, Perez-Diaz MD, Ceballo-Esparragon J, Jover-Navalon JM, Balibrea JM, Morales-Conde S (2020). Atencion dela urgencia quirurgica durante la pandemia COVID-19. Recomendaciones de la Asociation Espanola de Cirujanos. Cir Esp.

[5] Tebala GD, Lami M, Bond-Smith G (2021). Laparoscopic surgery and the coronavirus disease 2019 pandemic: a word from a different hymn-sheet. J Trauma Acute Care Surg.

[6] Castagneto-Gissey L, Casella G, Rossu MF, Del Corpo G, Iodice A, Lattina I, Ferrari P, Iannone I, Mongoli A, La Torre F (2020). Impact of COVID-19 outbreak on emergency surgery and emergency department admissions: an Italian level 2 emergency department experience. BJS.

[7] Palisi M, Massucco P, Mineccia M, Celano C, Giovanardi F, Ferrero A (2020). The disappearing of emergency surgery during the COVID 10 pandemic. Fact or fiction?. BJS.

[8] Tartaglia N, Pavone G, Lizzi V, Vovola F, Tricarico F, Pacilli M, Ambrosi A (2020). How emergency surgery has changed during the COVID-19 pandemic: a cohort study. Ann Med Surg.

[9] Fowler S, Zahir SF, Manning W, Kearney A, Sturgess D (2021). Effect of the COVID-19 pandemic first wave and public policy on elective and emergency surgery provision in Southern Queensland. ANZ J Surg.

[10] Nadell Farber O, Gomez GI, Titan AL, Fisher AT, Puntasecca CJ, Toro Arana V, Kempisky A, Wise CE, Bessoff KE, Hawn MT, Korndorffer JR, Forrester JD, Esquivel MM (2021). Impact of COVID-19 presentation, management and outcomes of acute care surgery for gallbladder disease and acute appendicitis. WJGS.

[11] Fallani G, Lombardi R, Masetti M, Chisari M, Zanini N, Cattaneo GM, Filosa M, Zanzi F, Guerra E, Bonilauri S, Di Donato L, Garulli G, Lucchi A, Grassia M, Ugolini G, Pasini F, Vetrone G, Benini C, Nicosia S, Jovine E (2021). Urgent and emergency surgery for secondary peritonitis during the COVID-19 outbreak: an unseen burden of a healthcare crisis. Upd Surg.

[12] De Simone B, Chouillard E, Di Saverio S, Pagani L, Sartelli M, Biffl WL, Coccolini F, Pieri A, Khan M, Borzellino G, Campanile FC, Ansaloni L, Catena F (2020). Emergency surgery during the COVID-19 pandemic: what you need to know for practice. Ann R Coll Surg Engl.

[13] Antunes D, Lami M, Chukwudi A, Dey A, Patel M, Shabana A, Shams M, Slack Z, Bond-Smith G, Tebala GD (2021). COVID-19 infection risk by open and laparoscopic surgical smoke: a systematic review of the Literature. Surgeon.

[14] Leong MQ, Lim CW, Lai YF (2021). Comparison of hospital-at-home models: a systematic review of reviews. BMJ Open.

[15] Ignat M, Philouze G, Aussenac-Belle L, Faucher V, Collange O, Mutter D, Pessaux P (2020). Small bowel ischaemia and SARS -CoV-2 infection: an underdiagnosed distinct clinical entity. Surgery.

[16] Norsa L, Valle C, Morotti D, Bonaffini PA, Indriolo A, Sonzogni A (2020). Intestinal ischaemia in the COVID-19 era. Dig Liver Dis.

